# Using Graphdiyne Nanoribbons for Molecular Electronics Spectroscopy and Nucleobase Identification: A Theoretical Investigation

**DOI:** 10.1021/acsaelm.3c01607

**Published:** 2024-02-01

**Authors:** M. Reza Rezapour, Blanca Biel

**Affiliations:** † Department of Atomic, Molecular and Nuclear Physics, Faculty of Science, Campus de Fuente Nueva, University of Granada, Granada 18071, Spain; ‡ Instituto Carlos I de Física Teórica y Computacional, University of Granada, Granada 18071, Spain

**Keywords:** graphdiyne, DNA sequencing, molecular electronics spectroscopy, biosensors, electron transport, Fano resonance

## Abstract

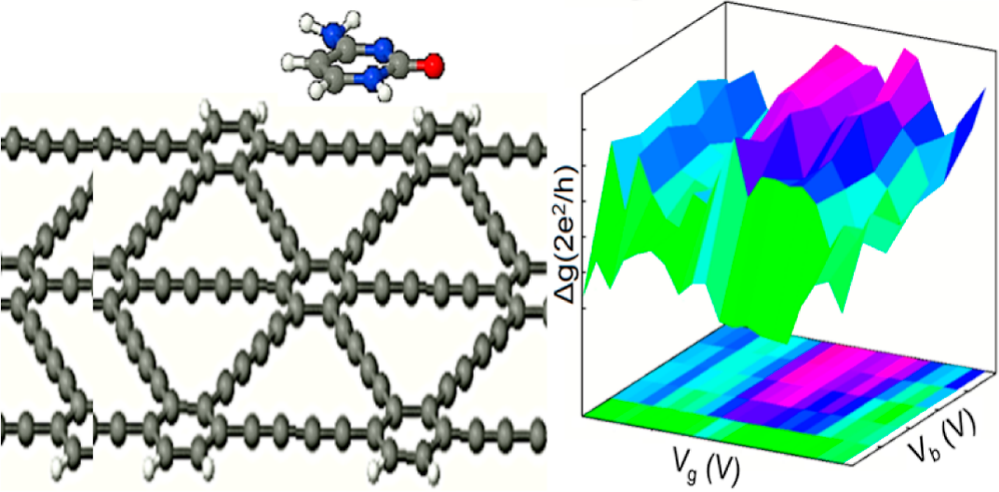

In pursuit of fast, cost-effective, and reliable DNA sequencing techniques, a variety of two-dimensional (2D) material-based nanodevices such as solid-state nanopores and nanochannels have been explored and established. Given the promising potential of graphene for the design and fabrication of nanobiosensors, other 2D carbon allotropes such as graphyne and graphdiyne have also attracted a great deal of attention as candidate materials for the development of sequencing technology. Herein, employing the 2D electronic molecular spectroscopy (2DMES) method, we investigate the capability of graphdiyne nanoribbons (GDNRs) as the building blocks of a feasible, precise, and ultrafast sequencing device. Using first-principles calculations, we study the adsorption of four canonical nucleobases (NBs), i.e., adenine (A), cytosine (C), guanine (G), and thymine (T) on an armchair GDNR (AGDNR). Our calculations reveal that compared to graphene, graphdiyne demonstrates more distinct binding energies for different NBs, indicating its more promising ability to unambiguously recognize DNA bases. Utilizing the 2DMES technique, we calculate the differential conductance (Δ*g*) of the studied NB–AGDNR systems and show that the resulting Δ*g* maps, unique for each NB–AGDNR complex, can be used to recognize each individual NB without ambiguity. We also investigate the conductance sensitivity of the proposed nanobiosensor and show that it exhibits high sensitivity and selectivity toward various NBs. Thus, our proposed graphdiyne-based nanodevice would hold promise for next-generation DNA sequencing technology.

## Introduction

2D materials have been a substantial focus of interest due to their remarkable structural, electrical, chemical, thermal, and optical characteristics.^1−8^ In the domain of layered materials, wonder material graphene and its one-dimensional (1D) form, graphene nanoribbon, have exhibited diverse applications across multiple sectors, ranging from electronics to energy, health, and the environment.^9−21^ Following the discovery of graphene, plenty of new carbon-based 2D materials such as graphyne, graphdiyne, graphone, and graphane have also been proposed and unveiled.^22,23^ The exceptional and distinctive characteristics of these novel materials position them as highly promising candidates for utilization across various nanotechnology domains.^24^ In particular, the significance of broadening the utilization of these materials in molecular sensing and DNA/RNA sequencing for the advancement of new techniques and devices^25−28^ makes the investigation of the interaction between biological molecules such as DNA and RNA and these carbon-based nanostructures particularly intriguing.

As new forms of non-natural carbon allotropes related to graphene, graphyne and graphdiyne have been the subject of interest due to their unique structure and intriguing electronic, optical, and mechanical properties, as well as their promising applications in nanoelectronics and energy storage.^29−31^ Graphyne and graphdiyne are allotropes of carbon with structures of one atom-thick planar sheets of sp- and sp^2^-bonded carbon atoms arranged in a crystal lattice.^32^ It has been shown that graphdiyne containing diacetylene linkages is the most stable non-natural carbon allotrope^33^ with high carrier mobility at room temperature (10^4^ to 10^5^ cm^2^ V^–1^ s^–1^).^34^ Unlike graphene, graphdiyne is a semiconductor with a natural band gap^35^ that facilitates its application in manufacturing nanoelectronic and photoelectronic devices. The minimal band gaps of graphdiyne have been predicted to fall within the range of 0.46–1.22 eV, depending on the methods employed and exchange–correlation functionals used.^36^ It is worth noting that accurate predictions of graphdiyne band gap can be achieved through GW or hybrid density functional theory (DFT) calculations.^37,38^ Graphdiyne also exhibits relatively lower stiffness compared to graphene which along with its intrinsic nonzero and readily tunable band gap paves the way toward its applications in the fabrication of a myriad of flexible electronic devices.^39^ Given that patterning the sheet structure into a nanoribbon is an established approach for adjusting the band gap of a material, 1D forms of graphdiyne have also been studied. It has been shown that both armchair graphdiyne nanoribbons (AGDNRs) and zigzag GDNRs (ZGDNRs) exhibit semiconducting behavior with their band gaps diminishing as the width of the ribbon increases.^40^ A theoretical investigation conducted by Bai et al.^41^ on GDNRs using the self-consistent field crystal orbital (SCF-CO) method under the periodic boundary conditions revealed that GDNRs exhibit greater stability in terms of energy compared to a 2D graphdiyne slab, with the stability diminishing as their widths increase. The mobilities of the GDNRs are also predicted to be in the range from 10^2^ to 10^6^ cm^2^ V^–1^ s^–1^ at room temperature, based on the effective mass approach and deformation potential theory. Thus, overlay GDNRs appear to be promising candidate materials for nanoelectronics applications.

On the other hand, the two-dimensional molecular electronic spectroscopy (2DMES) method, as detailed in our prior study,^42^ has proven highly efficient in differentiating various molecules, including DNA/RNA bases and their altered structures.^43^ This approach involves observing distinct reductions in the transmission profile of a system, caused by Fano resonance,^44^ once a molecule adheres to a monolayer. These specific Fano dips act as distinctive electronic signatures for the absorbed molecule, offering potential applications in molecular sensing and DNA/RNA sequencing. In line with the implementation of this technique and in order to avoid any ambiguity in the recognition of adsorbed molecules, the relative differential conductance (Δ*g*) of the molecule–monolayer system, unique to each complex, is mapped against bias and gate voltages.

Inspired by these findings, in this study, we investigate the potential of the AGDNR as a building block for a nanobiosensor designed for DNA sequencing purposes. To this end, first, we investigate the adsorption of four DNA bases, i.e., adenine (A), cytosine (C), guanine (G), and thymine (T) onto the AGDNR as well as the thermodynamic stability of the obtained NB–AGDNR complexes. Next, we calculate and plot transmission profiles of the energetically favored NB–AGDNR configurations and show that the transmission reductions that emerged at certain energies can be uniquely assigned to each nucleobase as their molecular electronic fingerprints. As the conductance of a biosensor is the measured quantity in practice, to ensure unambiguous recognition of different NBs in practical applications, we employ the 2DMES technique to generate Δ*g* of the spectrum of each NB–AGDNR system within a certain range of bias and gate voltages. We also show that the AGDNR exhibits significant and distinct conductance sensitivities across various NBs highlighting it as a functional material for DNA sequencing.

## Methods

DFT is employed to conduct the performed calculations. Relaxation calculations of all the geometries as well as investigation of the electronic structures of the systems are done using the Vienna ab initio simulation Package (VASP).^45^ Initially, the atomic structures of four nucleobases (NBs) and the pristine AGDNR underwent relaxation, followed by optimization of each NB–AGDNR system. The exchange–correlation effects were treated using the Perdew, Burke, and Ernzerhof^46^ generalized gradient approximation. Electron–ion interactions were described using the plane-augmented wave method, with Kohn and Sham orbitals expanded in a plane wave basis set.^47^ Given that van der Waals (vdW) forces^48^ rule the NB–AGDNR interaction in the proposed system, we incorporate a functional that addresses dispersion effects, reflecting these forces using the approach established by Tkatchenko–Scheffler (TS),^49^ which has been applied within the VASP framework.^50^ A real space integration mesh cutoff of 500 Ry, along with a double-ζ polarized (DZP) basis set, is employed in the calculations. The Brillouin zone is sampled with a mesh of 1 × 4 × 4 along the *x*, *y*, and *z* directions, respectively. All structures undergo complete relaxation until the energy and forces reach convergence values of 10^–5^ eV and 0.01 eV Å^–1^, respectively. The DFT method, in conjunction with nonequilibrium Green’s function,^51,52^ implemented in TranSIESTA,^53^ is employed to examine the transport properties of the systems. The same basis set, mesh cutoff, and functional utilized in relaxation processes are consistent with those employed in transport calculations. The *K*-grid points for both electrodes and the device are set to 1 × 1 × 64. The transmission coefficients of the studied systems are given by the following equation
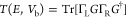
1where *V*_b_ represents the bias voltage, Tr denotes the trace, Γ_L/R_ = i[*Σ*_L/R_ – Σ_L/R_^†^] with Σ_L/R_ being the self-energy of the left/right electrode, and *G* = [*E* – *H* – *Σ*_L_ – Σ_R_]^−1^ represents Green’s function with the scattering region Hamiltonian *H*. Following the Landauer–Büttiker formalism, the source-drain current is given by
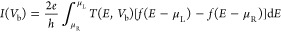
2where *f*(*E*, μ_L_) is the Fermi–Dirac function with the associated chemical potential of , representing a shifted value relative to the Fermi level (*E*_F_) of the neutral system. *E*_F_ changes upon the application of the gate voltage (*V*_g_), implying the virtual sweeping of [*f*(*E* – μ_L_) – *f*(*E* – μ_R_)] to a given energy point.

## Results and Discussion

**Figure 1:**
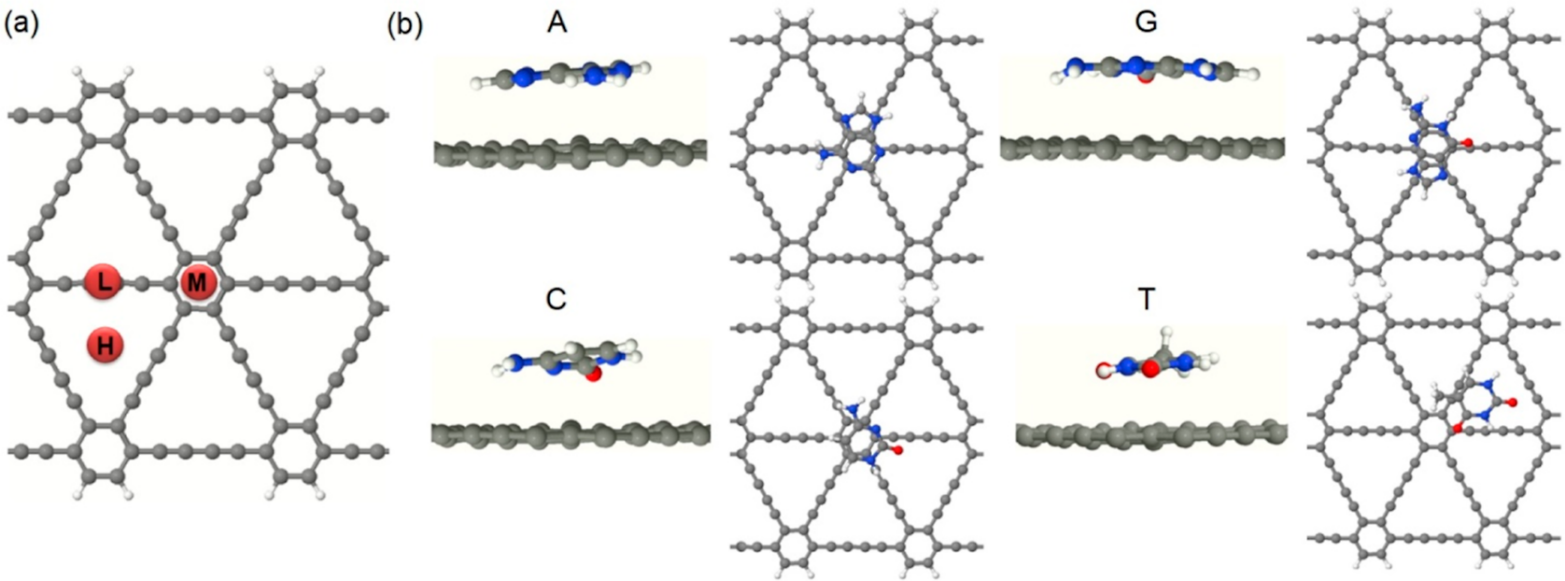
(a) Schematic representation of a 3-AGDNR. Adsorption sites of NBs over benzene moiety (M), acetylenic linkages (L), and hallow (H) are presented in maroon. (b) Fully relaxed geometries of NB–AGDNR configurations.

To conduct the study, we choose a 3-AGDNR in which 3 signifies the count of hexagonal carbon ring chains spanning the width of the AGDNR. To simplify, 3-AGDNR will be referred to as AGDNR. Initially, each NB is placed at the middle of the AGDNR on three different adsorption sites aligning parallel to the AGDNR surface at a vertical distance ranging from 2 to 3.5 Å. The separation distance (*d*) between the NB and AGDNR is defined as the distance between the closest atoms within the two subsystems. The three adsorption sites (Figure 1a), namely, acetylenic linkages (L), benzene moiety (M), and hollow (H), are chosen based on the structural symmetry of graphdiyne. The energetically favored configurations of the studied NB-AGDNR complexes are shown in Figure 1b. One can see that while the energetically suitable adsorption sites for A, C, and G are in the region of the benzene moiety, for T, the hollow region is more favored. This might result from the interaction of CO and CH_3_ groups of T with the π-clouds of graphdiyne.^54^

It is inferred from the depicted configurations that in all the structures, NBs take relatively tilted orientations toward the AGDNR surface which is slightly deformed at the adsorption site.

To investigate the relative stability of NBs on different adsorption sites, we calculate the binding energy (*E*_b_) of each NB onto the AGDNR’s surface for various *d* values using the following equation
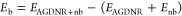
3where *E*_AGDNR+nb_, *E*_AGDNR_, and *E*_nb_ represent the total energies of the fully relaxed NB–AGDNR system, pristine AGDNR, and the isolated NB, respectively. Table 1 represents the binding energies of NBs adsorbed on different sites on the AGDNR along with the corresponding *d* value for each configuration. As is evident from the calculated *E*_b_ values, the adsorption process is exothermic, indicating the stability of systems. The binding strengths of the NBs with the AGDNR follow the order of G > A > C > T, which is in agreement with previous studies.^54,55^ It is worth noting that this sequence for graphene is G > A ≈ T ≈ C.^56^ Based on the calculated *E*_b_ and *d* values, it can be deduced that the strength of the interaction between the AGDNR and NB is determined by the separation distance *d* where G having the smallest *d* interacts relatively stronger with the AGDNR while T with the largest *d* shows the weakest interaction. The average *d* value for NB–AGDNR systems is about 3.2 Å which is in agreement with the sum of the vdW radii of C/O, C/N, and C/C.^57^ Therefore, the interaction between the NBs and AGDNR is nonbonded and the nature of the interaction is physisorption, leading to a small change in the structures of the two fragments after absorption. It is also worth mentioning that due to the involvement of weak vdW forces in the interaction between the NB and the substrate, the changes in *E*_b_ among different configurations of NB–AGDNR are expected to be negligible, regardless of the orientations of the NBs.^58^

**Table 1:** Binding Energies (*E*_b_) of NBs onto the AGDNR’s Surface on the Energetically Preferred Adsorption Sites Along With the Corresponding Separation Distance (*d*) between the NBs and AGDNR

		*E*_b_ (eV)	*d* (Å)
A	H	–0.57	2.89
	L	–0.59	2.89
	M	–0.61	2.87
C	H	–0.54	2.93
	L	–0.55	2.92
	M	–0.58	2.90
G	H	–0.63	2.85
	L	–0.66	2.84
	M	–0.68	2.82
T	H	–0.52	2.91
	L	–0.49	2.90
	M	–0.50	2.89

For further investigation of the mechanism of the interaction between the NBs and AGDNR, the charge density redistribution of the studied systems is calculated and plotted. The isosurfaces depicted in Figure S1 of the Supporting Information demonstrate that, across all the studied NB–AGDNR systems, the predominant concentration of the final charge density lies within the NB, indicating a lack of significant charge density redistribution between the NBs and AGDNR. This clearly indicates that the interaction between the two components of the system is regulated by weak and exclusively noncovalent forces, thus confirming the adsorption of NBs to the AGDNR surface.

**Figure 2:**
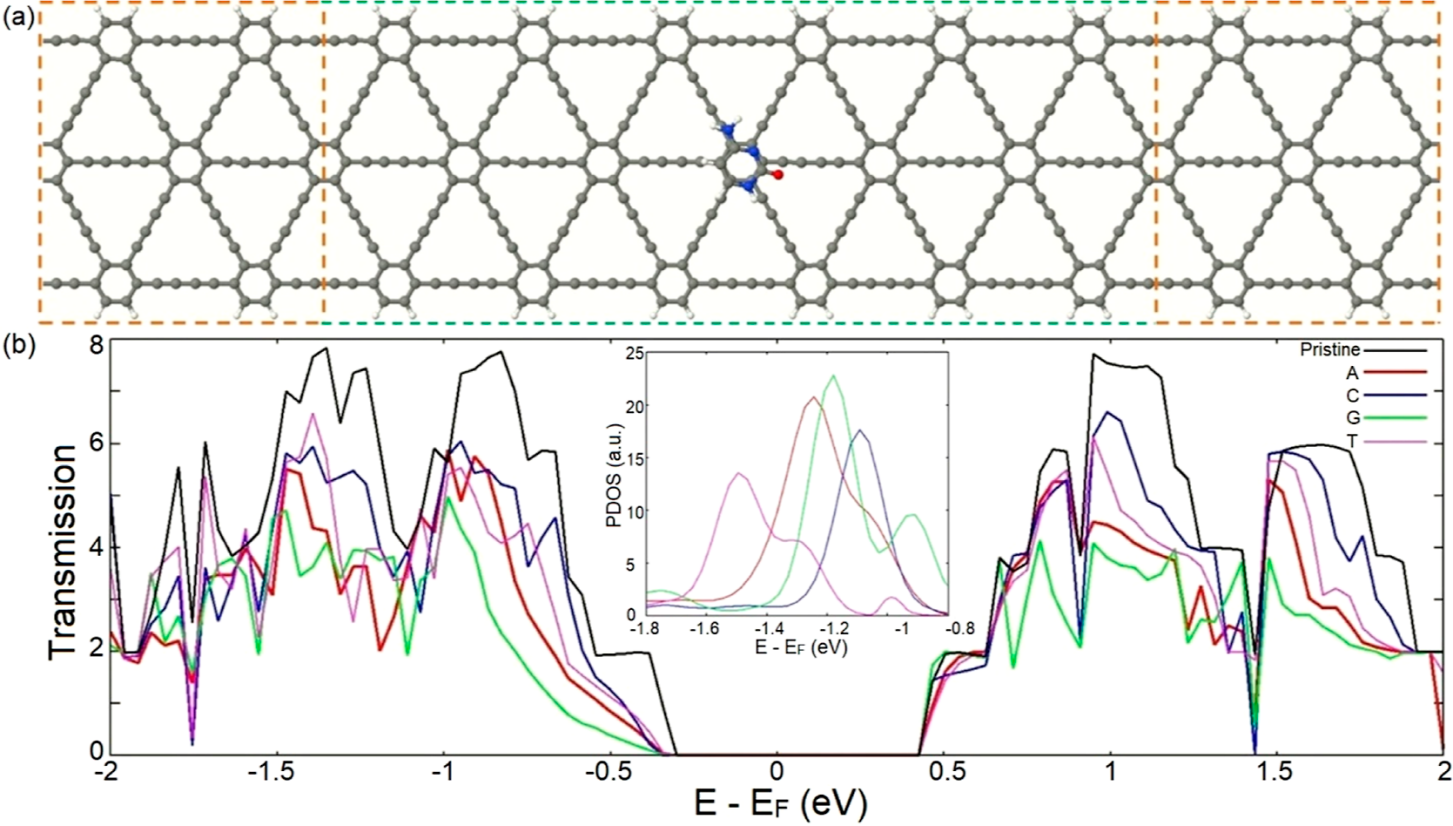
(a) Schematic illustration of the introduced two-probe transport system consisting of the AGDNR with an NB adsorbed on it. The electrodes and the scattering region are denoted by orange and green dashed lines, respectively. (b) Transmission profiles of the pristine AGDNR and NB–AGDNRs structures at zero *V*_b_. The inset depicts the PDOS of NB–AGDNR systems with respect to the DOS of the pristine AGDNR.

In the following, we investigate the charge transport characteristics of the introduced NB–AGDNR systems. Figure 2a illustrates a schematic representation of the introduced two-probe transport setup, which can be conceptualized as a Fano–Anderson model.^59^ Therefore, Fano peaks and dips are expected to be observed in the transmission profiles of NB–AGDNR structures due to Fano resonance. The calculated transmission profiles of the introduced NB–AGDNR complexes along with that of the pristine AGDNR at zero bias voltage (*V*_b_) are depicted in Figure 2b.

One can see from Figure 2b that the adsorption of an NB on the AGDNR leads to emergence of sharp reductions in the transmission spectrum of the AGDNR. The transmission profiles indicate that adsorption of A, C, G, and T on the AGDNR give rise to Fano dips at *E* – *E*_F_ = −1.19, −1.07 (as well as −1.68), −1.11, and −1.27 eV, respectively. As the charge transport characteristics of an NB adsorbed onto the surface of the AGDNR are significantly influenced by the energy states of the frontier molecular orbitals (MO), the observed dips in the transmission profiles can be distinctly associated with each NB. Thus, these dips can be utilized for molecular identification and DNA sequencing purposes. This assertion is also supported by analyzing the projected density of states (PDOSs) of the NB–AGDNR systems (plotted with respect to DOS of the pristine AGDNR), as depicted in the inset of Figure 2. The provided PDOS plots demonstrate that the adsorption of NBs induces strong states into the AGDNR’s electronic structure at energy values corresponding to the emerging transition reductions. It is noteworthy that the distinction between the energy values of the observed molecular fingerprints in the plotted transmission spectra provides an opportunity to distinguish different NBs unambiguously.

It also important to highlight that, since *E*_b_ in an NB–AGDNR system signifies the magnitude of the coupling parameter in the Fano–Anderson model,^44,59^ larger and more distinct binding energies of the NB on the AGDNR lead to more detectable transmission reductions or equivalently more distinguishable molecular fingerprints of NBs which increases the sensitivity of the introduced sequencing device in addition to reducing environmental and thermodynamic noise.

However, it can be contended that owing to the thermodynamic fluctuations of the NBs over the AGDNR, some molecular fingerprints mentioned earlier may fall within a narrow energy range, posing challenges in their distinction. In the following, we will show that the application of the 2DMES technique prevents possible unambiguity in recognizing NBs.

**Figure 3:**
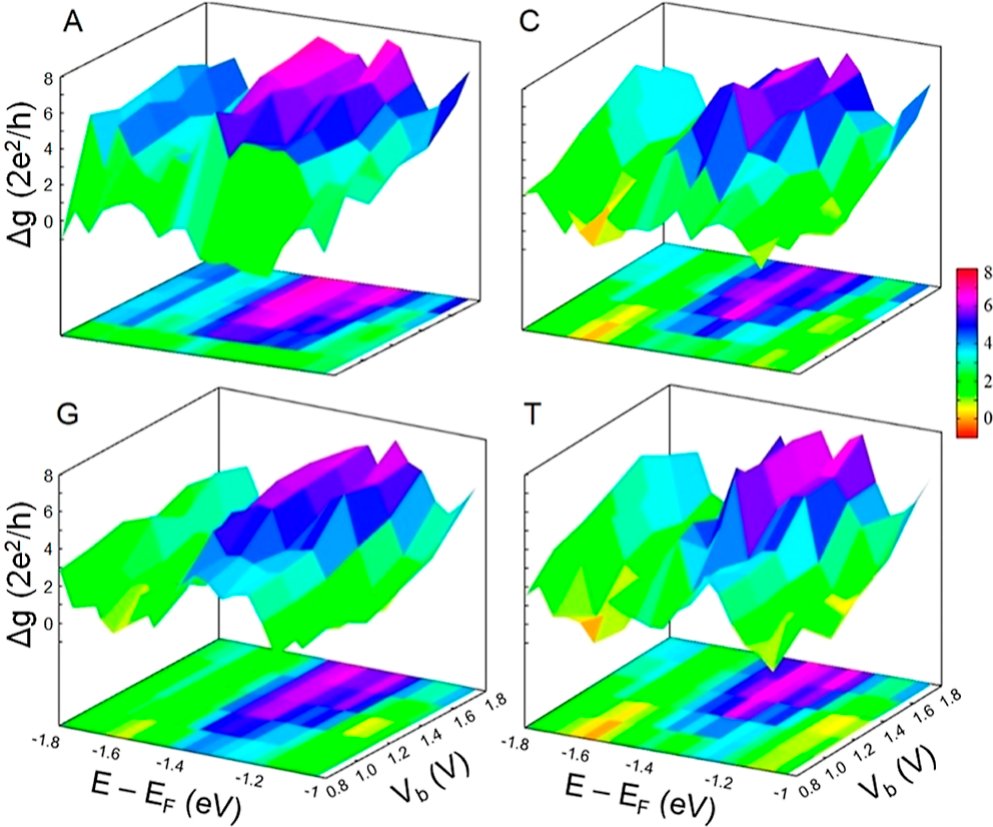
2D and 3D maps of differential conductance (Δ*g*) of the studied NB–AGDNR complexes against the electron channel energy (*E* – *E*_F_) and bias voltage *V*_b_.

As the practical measurement requires assessing the conductance of a biosensor, we calculate Δ*g* of the introduced sensing device under different *V*_b_ and *V*_g_ (or equivalently *E* – *E*_F_). It is also worth noting that since 1D conductance varies by a unit of quantum conductance, measuring it, as opposed to measuring current, offers a more accurate means of distinguishing changes in biosensor signals. Figure 3 depicts 2D and three-dimensional (3D) illustrations of the calculated Δ*g* maps with respect to *V*_b_ and *V*_g_ for different NB–AGDNR complexes. The graphs clearly show that the variations of the plotted Δ*g* surfaces for different studied systems are distinct.

.

This ensures that even in the case of two NBs providing Fano dips at adjacent energy values, their Δ*g* maps in the vicinity of their molecular fingerprints are recognizably different, allowing their unambiguous identification and differentiation. To gain more comprehensive insights into the better capability of the 2DMES method over the conventional current–voltage (*I*–*V*) measurement, we plot the *I*–*V* curves of the four NB–AGDNR systems as represented in Figure S2. One can see that the provided *I*–*V* graphs of the studied systems are hardly differentiable; hence, they are unsuitable for unambiguous distinction of different NBs.

Our proposed method is advantageous from a practical point of view as well. The time required for the measurement is the number of selected bias voltages multiplied by the time required for a gate voltage sweep. However, in the 2DMES method, there is no need to apply many bias voltages; only measurements at two or three different bias voltages would be sufficient to unambiguously distinguish NBs. This means that the time needed to conduct an accurate measurement is of the same order as that when the bias voltage is not controlled. On the other hand, scanning electron transport channels within a relatively narrow energy range, up to 1 eV (as evident from Δ*g* maps), is sufficient to generate a Δ*g* map for each nuclear interval, encompassing its distinct and individual electronic fingerprints. In practice, Δ*g* spectra corresponding to different molecule–nanoribbon arrangements can be stored in a database. Subsequently, by conducting data search and analysis, it becomes feasible to identify a molecule based on the acquired Δ*g* of spectroscopy data.

**Figure 4:**
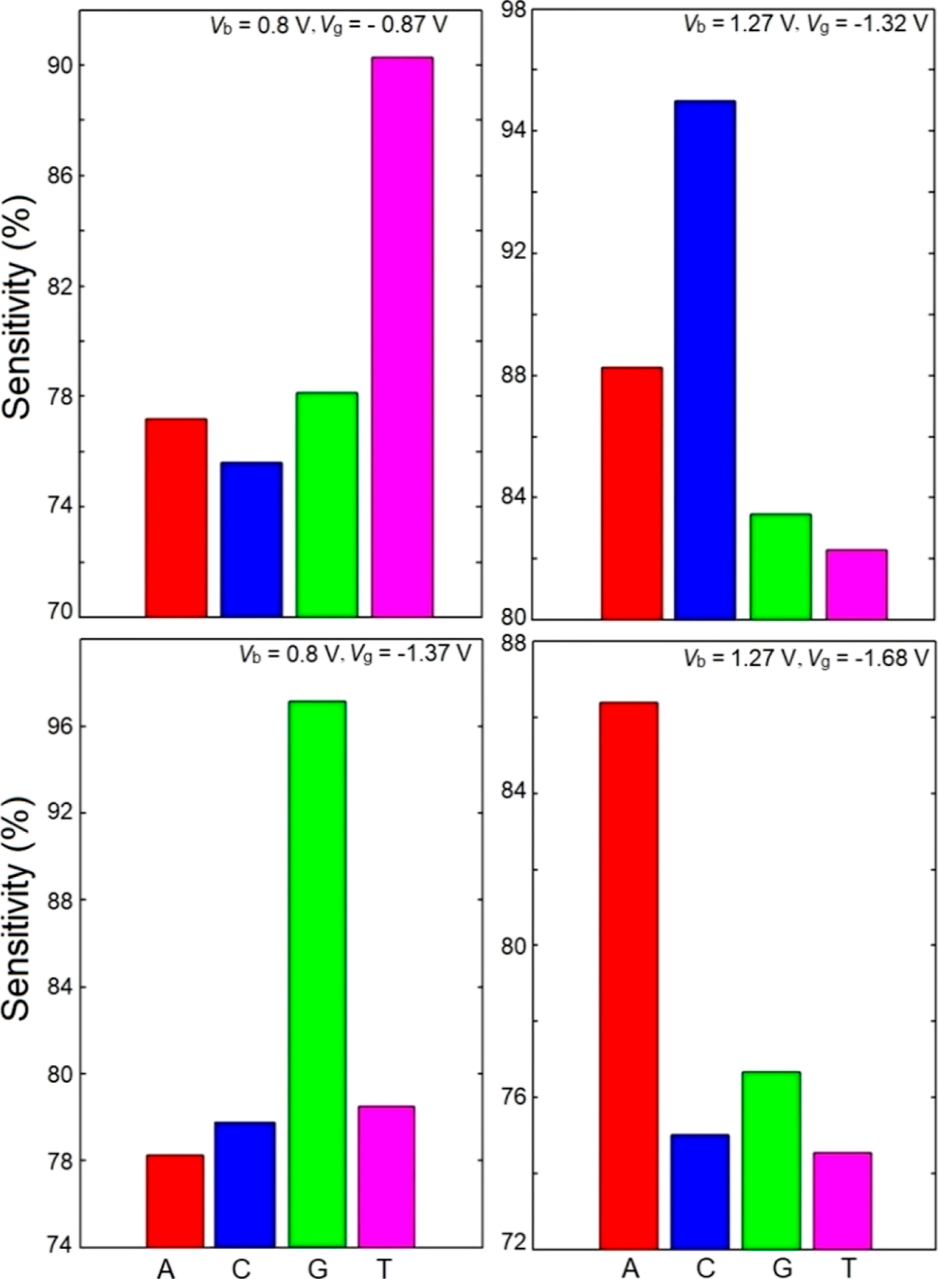
Sensitivity histograms of the AGDNR for different NBs at various *V*_b_ and *V*_g_ values.

To provide a practical example for the adequacy of the 2DMES technique in recognition of NBs unambiguously, we calculate the conductance sensitivity (*S*) of the studied systems using the following equation
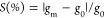
4where *g*_m_ and *g*_0_ are the conductance of the introduced biosensor with and without the adsorbed molecule, respectively. The histograms plotted in Figure 4 represent the sensitivity values for the studied NB–AGDNR systems calculated with four different combinations of *V*_b_ and *V*_g_. One can see that the variation of *S* for different NBs can be drastic when *V*_b_ and *V*_g_ are swept. For instance, while at *V*_b_ = 0.8 V, *S* is larger for T (≈90%) and G (≈98%) at *V*_g_ = −0.87 V and *V*_g_ = −1.37 V, respectively, at *V*_b_ = 1.27 V, the device shows higher ***S*** for C (≈95%) and A (≈86%) at *V*_g_ = −1.32 V and *V*_g_ = −1.68 V, respectively. It is also clear from the histograms that the order of the sensitivity values for different NBs changes in different combinations of *V*_b_ and *V*_g_.

It should be noted that in a more realistic picture where the device is used in aqueous solution, water molecules may affect the performance of the device. Feliciano et al. showed that small changes are observed in the transmission plots due to the screening effect assisted by the water molecules.^60^ However, they also concluded that the presence of water only affects the magnitude of the measured current, and hence the trend observed within systems does not change. A more detailed discussion regarding the environmental noise and the response time and the stability of the device is presented in the Supporting Information. It also has been shown that the transmission functions in the energy range of interest, hence the provided Δ*g* maps, are not affected significantly in response to environmental effects such as backbones, hydrated phosphate groups, and counterions.^61^ Thus, the proposed graphdiyne-based sequencing device possesses a notable capability to distinctly identify different NBs via using the measured conductance data at various *V*_b_ and *V*_g_ values through the 2DMES method.

## Conclusions

A detailed computational study is performed to investigate the single molecular recognition capability of armchair type graphdiyne nanoribbons via employing the 2DMES technique for DNA sequencing purposes. To this end, we initially study the adsorption behavior of four DNA bases, i.e., adenine, cytosine, guanine, and thymine, onto an AGDNR. Our calculations reveal the noncovalent physisorption of the NBs onto the AGDNR’s surface follows the order of G > A > *T* > C in terms of the binding energy magnitudes, which is more pronounced compared to that observed for graphene (G > A ≈ T ≈ C). By calculating transmission spectra of NB–AGDNR systems, we show that adsorbed NBs on the AGDNR’s surface generate sharp reductions in distinct energies in the transmission spectrum of the AGDNR that can be uniquely assigned to each NB and hence employed to identify the adsorbed molecule. To eliminate ambiguity in the identification of each individual NB and also offer a feasible method to employ the proposed device in practice, we provide differential conductance maps of the introduced complexes by sweeping both bias and gate voltages applied to the systems. In contrast to the 1D current–voltage profiles which hardly distinguish different molecules, the provided 2D and 3D Δ*g* maps, which can be stored in a data set, show distinct features for different NB–AGDNR systems which enable the unambiguous identification of various NBs. The admissible susceptibility of the introduced AGDNR-based biosensor is also examined by calculating its conductance sensitivity for different DNA bases. It is shown that the AGDNR exhibits significant and discernible sensitivities across various NBs at different *V*_b_ and *V*_g_ values, rendering it appropriate for applications in molecular sensing.

## Supporting Information Available

Charge density redistribution and current–voltage profiles (PDF)

## References

[ref1] Khan K.; Tareen A. K.; Aslam M.; Wang R.; Zhang Y.; Mahmood A.; Ouyang Z.; Zhang Z.; Zhang H.; Zhang Z. Recent Developments in Emerging Two-Dimensional Materials and Their Applications. J. Mater. Chem. C 2020, 8, 387–440. 10.1039/c9tc04187g.

[ref2] Zeng M.; Xiao Y.; Liu J.; Yang K.; Fu L.; Fu K. Exploring Two-Dimensional Materials toward the Next-Generation Circuits: From Monomer Design to Assembly Control. Chem. Rev. 2018, 118, 6236–6296. 10.1021/acs.chemrev.7b00633. 29381058

[ref3] Liu C.; Chen H.; Wang S.; Liu Q.; Jiang Y. G.; Zhang D. W.; Liu M.; Zhou P. Two-Dimensional Materials for Next-Generation Computing Technologies. Nat. Nanotechnol. 2020, 15, 545–557. 10.1038/s41565-020-0724-3. 32647168

[ref4] Akinwande D.; Huyghebaert C.; Wang C. H.; Serna M. I.; Goossens S.; Li L. J.; Wong H. S. P.; Koppens F. H. L. Graphene and Two-Dimensional Materials for Silicon Technology. Nature 2019, 573, 507–518. 10.1038/s41586-019-1573-9. 31554977

[ref5] Biel B.; Blase X.; Triozon F.; Roche S. Anomalous Doping Effects on Charge Transport in Graphene Nanoribbons. Phys. Rev. Lett. 2009, 102, 096803 10.1103/PhysRevLett.102.096803. 19392549

[ref6] Cresti A.; Nemec N.; Biel B.; Niebler G.; Triozon F.; Cuniberti G.; Roche S. Charge Transport in Disordered Graphene-Based Low Dimensional Materials. Nano Res. 2008, 1, 361–394. 10.1007/s12274-008-8043-2.

[ref7] Lherbier A.; Biel B.; Niquet Y. M.; Roche S. Transport Length Scales in Disordered Graphene-Based Materials: Strong Localization Regimes and Dimensionality Effects. Phys. Rev. Lett. 2008, 100, 036803 10.1103/PhysRevLett.100.036803. 18233020

[ref8] Mittal S.; Manna S.; Jena M. K.; Pathak B. Decoding Both DNA and Methylated DNA Using a MXene-Based Nanochannel Device: Supervised Machine-Learning-Assisted Exploration. ACS Mater. Lett. 2023, 5, 1570–1580. 10.1021/acsmaterialslett.3c00117.

[ref9] Ferrari A. C.; Bonaccorso F.; Fal’ko V.; Novoselov K. S.; Roche S.; Bøggild P.; Borini S.; Koppens F. H. L.; Palermo V.; Pugno N.; Garrido J. A.; Sordan R.; Bianco A.; Ballerini L.; Prato M.; Lidorikis E.; Kivioja J.; Marinelli C.; Ryhänen T.; Morpurgo A.; Coleman J. N.; Nicolosi V.; Colombo L.; Fert A.; Garcia-Hernandez M.; Bachtold A.; Schneider G. F.; Guinea F.; Dekker C.; Barbone M.; Sun Z.; Galiotis C.; Grigorenko A. N.; Konstantatos G.; Kis A.; Katsnelson M.; Vandersypen L.; Loiseau A.; Morandi V.; Neumaier D.; Treossi E.; Pellegrini V.; Polini M.; Tredicucci A.; Williams G. M.; Hee Hong B.; Ahn J.-H.; Min Kim J.; Zirath H.; van Wees B. J.; van der Zant H.; Occhipinti L.; Di Matteo A.; Kinloch I. A.; Seyller T.; Quesnel E.; Feng X.; Teo K.; Rupesinghe N.; Hakonen P.; Neil S. R. T.; Tannock Q.; Löfwander T.; Kinaret J. Science and Technology Roadmap for Graphene, Related Two-Dimensional Crystals, and Hybrid Systems. Nanoscale 2015, 7, 4598–4810. 10.1039/c4nr01600a. 25707682

[ref10] Pan Z. H.; Liu N.; Fu L.; Liu Z. F. Wrinkle Engineering: A New Approach to Massive Graphene Nanoribbon Arrays. J. Am. Chem. Soc. 2011, 133, 17578–17581. 10.1021/ja207517u. 21981554

[ref11] Thomas S.; Rajan A. C.; Rezapour M. R.; Kim K. S. In Search of a Two-Dimensional Material for DNA Sequencing. J. Phys. Chem. C 2014, 118 (20), 10855–10858. 10.1021/jp501711d.

[ref12] Muñoz-Rojas F.; Fernández-Rossier J.; Palacios J. J. Giant Magnetoresistance in Ultrasmall Graphene-Based Devices. Phys. Rev. Lett. 2009, 102, 136810 10.1103/PhysRevLett.102.136810. 19392393

[ref13] Rezapour M. R.; Yun J.; Lee G.; Kim K. S. Lower Electric Field-Driven Magnetic Phase Transition and Perfect Spin Filtering in Graphene Nanoribbons by Edge Functionalization. J. Phys. Chem. Lett. 2016, 7 (24), 5049–5055. 10.1021/acs.jpclett.6b02437. 27973868

[ref14] Leconte N.; Soriano D.; Roche S.; Ordejon P.; Charlier J. C.; Palacios J. J. Magnetism-Dependent Transport Phenomena in Hydrogenated Graphene: From Spin-Splitting to Localization Effects. ACS Nano 2011, 5, 3987–3992. 10.1021/nn200558d. 21469688

[ref15] Jiang X.; Nisar J.; Pathak B.; Zhao J.; Ahuja R. Graphene Oxide as a Chemically Tunable 2-D Material for Visible-Light Photocatalyst Applications. J. Catal. 2013, 299, 204–209. 10.1016/j.jcat.2012.12.022.

[ref16] Rezapour M. R.; Lee G.; Kim K. S. An Effective Approach to Realize Graphene Based p-n Junctions via Adsorption of Donor and Acceptor Molecules. Carbon 2019, 153, 525–530. 10.1016/j.carbon.2019.07.062.

[ref17] Choudhuri I.; Patra N.; Mahata A.; Ahuja R.; Pathak B. B-N@ graphene: Highly Sensitive and Selective Gas Sensor. J. Phys. Chem. C 2015, 119, 24827–24836. 10.1021/acs.jpcc.5b07359.

[ref18] Avdoshenko S. M.; Nozaki D.; Gomes da Rocha C.; González J. W.; Lee M. H.; Gutierrez R.; Cuniberti G. Dynamic and Electronic Transport Properties of DNA Translocation through Graphene Nanopores. Nano Lett. 2013, 13, 1969–1976. 10.1021/nl304735k. 23586585

[ref19] Kim H. S.; Kim Y. H. Recent Progress in Atomistic Simulation of Electrical Current DNA Sequencing. Biosens. Bioelectron. 2015, 69, 186–198. 10.1016/j.bios.2015.02.020. 25744599

[ref20] Biel B.; Triozon F.; Blase X.; Roche S. Chemically Induced Mobility Gaps in Graphene Nanoribbons: A Route for Upscaling Device Performances. Nano Lett. 2009, 9, 2725–2729. 10.1021/nl901226s. 19530669

[ref21] Rezapour M. R.; Lee G.; Kim K. S. A High Performance N-doped Graphene Nanoribbon Based Spintronic Device Applicable with a Wide Range of Adatoms. Nanoscale Adv. 2020, 2, 5905–5911. 10.1039/D0NA00652A. 36133856 PMC9419213

[ref22] Jana S.; Bandyopadhyay A.; Datta S.; Bhattacharya D.; Jana D. Emerging Properties of Carbon based 2D Material Beyond Graphene. J. Phys.: Condens. Matter 2022, 34, 053001 10.1088/1361-648X/ac3075. 34663760

[ref23] Peng Q.; Crean J.; Han L.; Liu S.; Wen X.; De S.; Dearden A. New Materials Graphyne, Graphdiyne, Graphone, and Graphane: Review of Properties, Synthesis, and Application in Nanotechnology. Nanotechnol. Sci. Appl. 2014, 7, 1–29. 10.2147/NSA.S40324. 24808721 PMC3998860

[ref24] Srinivasu K.; Ghosh S. K. Graphyne and Graphdiyne: Promising Materials for Nanoelectronics and Energy Storage Applications. J. Phys. Chem. C 2012, 116, 5951–5956. 10.1021/jp212181h.

[ref25] Kumawat R. L.; Pathak B. Electronic Conductance and Current Modulation through Graphdiyne Nanopores for DNA Sequencing. ACS Appl. Electron. Mater. 2021, 3, 3835–3845. 10.1021/acsaelm.1c00452.

[ref26] Parvin N.; Jin Q.; Wei Y.; Yu R.; Zheng B.; Huang L.; Zhang Y.; Wang L.; Zhang H.; Gao M.; Zhao H.; Hu W.; Li Y.; Wang D. Few-Layer Graphdiyne Nanosheets Applied for Multiplexed Real-Time DNA Detection. Adv. Mater. 2017, 29, 1606755 10.1002/adma.201606755. 28295711

[ref27] Chen X.; Gao P.; Guo L.; Zhang S. Graphdiyne as a Promising Material for Detecting Amino Acids. Sci. Rep. 2015, 5, 16720 10.1038/srep16720. 26568200 PMC4644954

[ref28] Liu J.; Chen C.; Zhao Y. Progress and Prospects of Graphdiyne-Based Materials in Biomedical Applications. Adv. Mater. 2019, 31, 1804386 10.1002/adma.201804386. 30773721

[ref29] Huang C.; Li Y.; Wang N.; Xue Y.; Zuo Z.; Liu H.; Li Y. Progress in Research into 2D Graphdiyne-Based Materials. Chem. Rev. 2018, 118, 7744–7803. 10.1021/acs.chemrev.8b00288. 30048120

[ref30] Li H.; Lim J. H.; Lv Y.; Li N.; Kang B.; Lee J. Y. Graphynes and Graphdiynes for Energy Storage and Catalytic Utilization: Theoretical Insights into Recent Advances. Chem. Rev. 2023, 123, 4795–4854. 10.1021/acs.chemrev.2c00729. 36921251

[ref31] Narita N.; Nagai S.; Suzuki S.; Nakao K. Optimized Geometries and Electronic Structures of Graphyne and its Family. Phys. Rev. B 1998, 58, 11009–11014. 10.1103/PhysRevB.58.11009.

[ref32] Kang J.; Li J.; Wu F.; Li S. S.; Xia J. B. Elastic, Electronic, and Optical Properties of Two-Dimensional Graphyne Sheet. J. Phys. Chem. C 2011, 115, 20466–20470. 10.1021/jp206751m.

[ref33] Haley M. M.; Brand S. C.; Pak J. J. Carbon Networks Based on Dehydrobenzoannulenes: Synthesis of Graphdiyne Substructures. Angew. Chem., Int. Ed. Engl. 1997, 36, 836–838. 10.1002/anie.199708361.

[ref34] Kang J.; Wei Z.; Li J. Graphyne and its Family: Recent Theoretical Advances. ACS Appl. Mater. Interfaces 2019, 11 (3), 2692–2706. 10.1021/acsami.8b03338. 29663794

[ref35] Long M.; Tang L.; Wang D.; Li Y.; Shuai Z. Electronic Structure and Carrier Mobility in Graphdiyne Sheet and Nanoribbons: Theoretical Predictions. ACS Nano 2011, 5, 2593–2600. 10.1021/nn102472s. 21443198

[ref36] Ivanovskii A. L. Graphynes and graphdyines. Prog. Solid State Chem. 2013, 41, 1–19. 10.1016/j.progsolidstchem.2012.12.001.

[ref37] Luo G.; Qian X.; Liu H.; Qin R.; Zhou J.; Li L.; Gao Z.; Wang E.; Mei W. N.; Lu J.; et al. Quasiparticle Energies and Excitonic Effects of the Two-Dimensional Carbon Allotrope Graphdiyne: Theory and Experiment. Phys. Rev. B 2011, 84, 075439 10.1103/physrevb.84.075439.

[ref38] Pari S.; Cuellar A.; Wong B. M. Structural and Electronic Properties of Graphdiyne Carbon Nanotubes from Large-Scale DFT Calculations. J. Phys. Chem. C 2016, 120, 18871–18877. 10.1021/acs.jpcc.6b05265.

[ref39] Zhao Y.; Chai L.; Yan X.; Huang W.; Fan T.; Al-Hartomy O. A.; Al-Ghamdi A.; Wageh S.; Al-Sehemi A. G.; Xie Z.; Zhang H. Characteristics, Properties, Synthesis and Advanced Applications of 2D Graphdiyne versus Graphene. Mater. Chem. Front. 2022, 6, 528–552. 10.1039/D1QM01342D.

[ref40] Pan L. D.; Zhang L. Z.; Song B. Q.; Du S. X.; Gao H. J. Graphyne- and Graphdiyne-Based Nanoribbons: Density Functional Theory Calculations of Electronic Structures. Appl. Phys. Lett. 2011, 98, 173102 10.1063/1.3583507.

[ref41] Bai H.; Zhu Y.; Qiao W.; Huang Y. Structures, Stabilities and Electronic Properties of Graphdiyne Nanoribbons. RSC Adv. 2011, 1, 768–775. 10.1039/c1ra00481f.

[ref42] Rajan A. C.; Rezapour M. R.; Yun J.; Cho Y.; Cho W. J.; Min S. K.; Lee G.; Kim K. S. Two Dimensional Molecular Electronics Spectroscopy for Molecular Fingerprinting, DNA Sequencing, and Cancerous DNA Recognition. ACS Nano 2014, 8, 1827–1833. 10.1021/nn4062148. 24446806

[ref43] Reza Rezapour M.; Biel B. DNA/RNA Sequencing Using Germanene Nanoribbons via Two Dimensional Molecular Electronic Spectroscopy: An ab Initio Study. Nanoscale 2022, 14, 5147–5153. 10.1039/d1nr07336b. 35302137

[ref44] Miroshnichenko A. E.; Flach S.; Kivshar Y. S. Fano Resonances in Nanoscale Structures. Rev. Mod. Phys. 2010, 82, 2257–2298. 10.1103/RevModPhys.82.2257.

[ref45] Kresse G.; Furthmüller J. Efficient Iterative Schemes for ab Initio Total-Energy Calculations Using a Plane-Wave Basis Set. Phys. Rev. B 1996, 54, 11169–11186. 10.1103/PhysRevB.54.11169. 9984901

[ref46] Perdew J. P.; Burke K.; Ernzerhof M. Generalized Gradient Approximation Made Simple. Phys. Rev. Lett. 1996, 77, 3865–3868. 10.1103/PhysRevLett.77.3865. 10062328

[ref47] Kresse G.; Joubert D. From Ultrasoft Pseudopotentials to the Projector Augmented-Wave Method. Phys. Rev. B 1999, 59, 1758–1775. 10.1103/PhysRevB.59.1758.

[ref48] Björk J.; Hanke F.; Palma C. A.; Samori P.; Cecchini M.; Persson M. Adsorption of Aromatic and Anti-Aromatic Systems on Graphene through π-π Stacking. J. Phys. Chem. Lett. 2010, 1, 3407–3412. 10.1021/jz101360k.

[ref49] Tkatchenko A.; Scheffler M. Accurate Molecular Van Der Waals Interactions from Ground-State Electron Density and Free-Atom Reference Data. Phys. Rev. Lett. 2009, 102, 073005 10.1103/PhysRevLett.102.073005. 19257665

[ref50] Bučko T.; Lebègue S.; Hafner J.; Ángyán J. G. Tkatchenko-Scheffler van der Waals Correction Method with and without Self-Consistent Screening Applied to Solids. Phys. Rev. B 2013, 87, 064110 10.1103/PhysRevB.87.064110.

[ref51] Taylor J.; Guo H.; Wang J. Ab initio Modeling of Quantum Transport Properties of Molecular Electronic Devices. Phys. Rev. B 2001, 63, 245407 10.1103/PhysRevB.63.245407.

[ref52] Datta S. Electronic Transport in Mesoscopic Systems; Cambridge University Press: Cambridge, 1997.

[ref53] Brandbyge M.; Mozos J. L.; Ordejón P.; Taylor J.; Stokbro K. Density-Functional Method for Nonequilibrium Electron Transport. Phys. Rev. B 2002, 65, 165401 10.1103/PhysRevB.65.165401.

[ref54] Chandra Shekar S.; Swathi R. S. Stability of Nucleobases and Base Pairs Adsorbed on Graphyne and Graphdiyne. J. Phys. Chem. C 2014, 118, 4516–4528. 10.1021/jp412791v.

[ref55] Gao H.; Qiao W.; Zhu M.; Wu J.; Zhang X.; Yan W.; Wu Y.; Zhang H.; Bai H.; Li Y. First-Principle Insights into the Non-Covalent Interaction Between Nucleotide Bases and Flat Nanocarbon: Graphene vs Graphdiyne. Diamond Relat. Mater. 2023, 139, 110366 10.1016/j.diamond.2023.110366.

[ref56] Gowtham S.; Scheicher R. H.; Ahuja R.; Pandey R.; Karna S. P. Physisorption of Nucleobases on Graphene: Density-Functional Calculations. Phys. Rev. B: Condens. Matter Mater. Phys. 2007, 76, 033401 10.1103/PhysRevB.76.033401.

[ref57] Batsanov S. S. Van der Waals Radii of Elements. Inorg. Mater. 2001, 37, 871–885. 10.1023/A:1011625728803.

[ref58] de Oliveira I. S. S.; Miwa R. H. Organic Molecules Deposited on Graphene: A Computational Investigation of Self-Assembly and Electronic Structure. J. Chem. Phys. 2015, 142, 044301 10.1063/1.4906435. 25637981

[ref59] Rezapour M. R.; Rajan A. C.; Kim K. S. Molecular Sensing Using Armchair Graphene Nanoribbon. J. Comput. Chem. 2014, 35, 1916–1920. 10.1002/jcc.23705. 25117934

[ref60] Feliciano G. T.; Sanz-Navarro C.; Coutinho-Neto M. D.; Ordejón P.; Scheicher R. H.; Rocha A. R. Addressing the Environment Electrostatic Effect on Ballistic Electron Transport in Large Systems: A QM/MM-NEGF Approach. J. Phys. Chem. B 2018, 122, 485–492. 10.1021/acs.jpcb.7b03475. 28721724

[ref61] Min S. K.; Kim W. Y.; Cho Y.; Kim K. S. Fast DNA Sequencing with a Graphene-Based Nanochannel Device. Nat. Nanotechnol. 2011, 6, 162–165. 10.1038/nnano.2010.283. 21297626

