# Supplementary material for: Using Graphdiyne Nanoribbons for Molecular Electronics Spectroscopy and Nucleobase Identification: A Theoretical Investigation
Source: ACS Appl Electron Mater. 2024 Feb 1;6(2):1244–51. doi: 10.1021/acsaelm.3c01607 (PMC10902847; doi:10.1021/acsaelm.3c01607)
Supplement: Supplementary file 1 — el3c01607_si_001.pdf [file el3c01607_si_001.pdf]

Supporting Information

"Using Graphdiyne Nanoribbons for Molecular Electronics Spectroscopy and Nucleobase Identification: a Theoretical Investigation"

M. Reza Rezapour,*[a] Blanca Biel [ab]

[a]Department of Atomic, Molecular and Nuclear Physics, Faculty of Science, Campus de Fuente Nueva, University of Granada, 18071 Granada, Spain.

[b] Instituto Carlos I de Física Teórica y Computacional, University of Granada, 18071 Granada, Spain.

**rezapour@ugr.es*

Charge density redistribution

To provide a deeper insight into the nature of the interaction between nucleobases (NBs) and AGDNR, we plot isosurfaces of the calculated spatial charge density difference for all NBs adsorbed on AGDNR. For this purpose, the following equation is used to calculate the charge density change:

$$\Delta\rho(\vec{r}) = \rho_{AGeNR+base}(\vec{r}) - \rho_{AGeNR}(\vec{r}) - \rho_{base}(\vec{r}) \quad \text{(S1)}$$

where $\rho_{AGeNR+base}(\vec{r})$, $\rho_{AGeNR}(\vec{r})$, and $\rho_{base}(\vec{r})$ are the charge density of NB-AGDNR system, AGDNR, and NB respectively. Figure S1 illustrates the calculated charge density isosurfaces for the studied NB-AGDNR systems. It can be deduced from the plotted isosurfaces that there is no complex redistribution of charge density between NBs and AGDNR. This is also confirmed with our Mulliken and Bader charge analysis which shows a negligible average charge transfer of $0.012e_0$ between two fragments of the NB-AGDNR structures. This indicates that the interaction of NBs with AGDNR has the physisorption nature and is madiated by weak vdW forces. The obtained results are in agreement with previous studies (Ref. 55 in the main text).

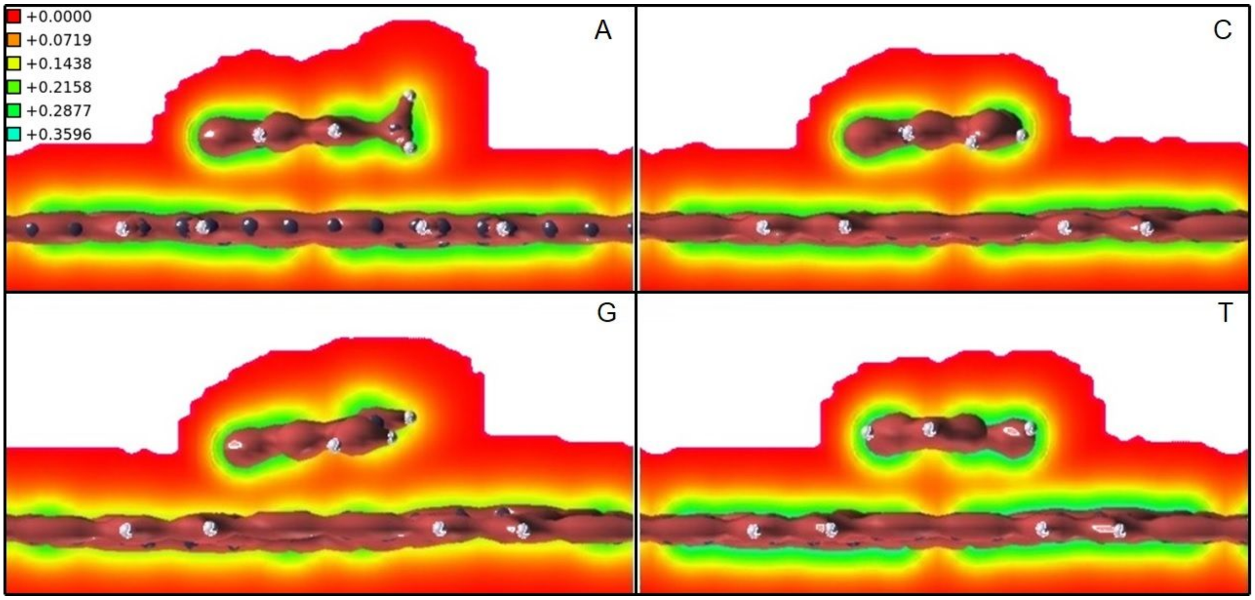

Figure S1: Spatial charge density redistribution of different NB-AGDNR systems.

Current-voltage profiles of NB-AGDNR systems

The calculated current-voltage (*I*-*V*) profile of the introduced NB-AGDNR systems is represented in Figure S2 with the aim of comparing the capabilities of 1D current measurement and 2DMES technique in unambiguous identification of various NBs. The analysis of the plotted *I*-*V* curves suggests that achieving a discernible distinction between NBs using 1D current measurement with acceptable resolution necessitates elevated bias voltages. This implies that relying solely on *I*-*V* measurement may prove insufficient for unequivocal identification of all NBs, particularly at low bias voltages. Therefore, it is required to sweep gate voltage along with bias voltage in a certain window to provide a conductance map for every NB, as is performed in 2DMES technique, for a high resolution molecular recognition.

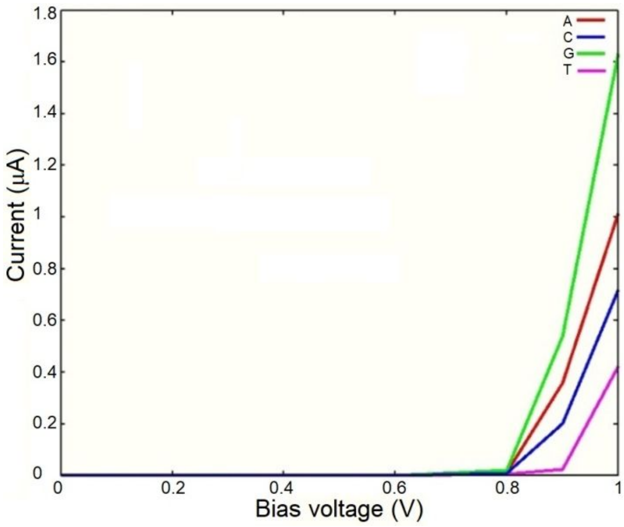

Figure S2: Current-voltage profiles of different NB-AGDNR systems.

Noise, Response Time, and Stability of the Device

In this section, we present a qualitative discussion on the noise, response time and stability of the introduced sequencing device.

Noise: The main source of noise in nanochannel based biosensors is fluctuations of molecules on the membrane's surface originated from ambient and/or thermodynamic effects. Since π−π stacking is the ruling interaction between NBs and AGDNR in the introduced system, it is expected that perturbation of the electric current towards the noise level would be significantly reduced due to large binding energy of NBs onto the AGDNR's surface. It is also noteworthy that such π-stacking is much stronger than the H–π interactions between water molecules and AGDNR, hence, the stacking structures are not significantly influenced by solvent effects. Moreover, since it is the conductance of the device that is measured in the 2DMES method, even a clearer distinction of the change in the signal of the device can be made because conductance through the device changes by a unit of quantum conductance G0 (= $2e^2/h$) in its interaction with NBs which is experimentally measurable and well above the noise level at room temperature.

Response Time: At each NB translocation step, the 2D conductance of AGDNR should be measured for a whole sweep of the chemical potential, which is controlled experimentally via a back-gate capacitively coupled to the AGDNR. Thus, the rate-determining step is the voltage sweep. The transit time of each

base should be controlled to be longer (~10 μs) than the gate voltage sweeping time (~0.1 μs, ~10 MHz) which may vary based on the advances in the present experimental capability.

Stability: Unlike biological nanopores that suffer from limited stability with respect to modifications in physical parameters and chemical conditions such as pH, temperature, and salt concentration,[1] solid-state nanopores and nanochannels exhibit high stability, tunable geometry, and controllable surface chemistry.[2] However, in the comparison between advantages and disadvantages of solid-state nanopores and nanochannels, it is worth noting that the size of nanopores may change during the sequencing processes.

REFERENCES:

1- S. Agah, M. Zheng, M. Pasquali, A. B. Kolomeisky, DNA Sequencing by Nanopores: Advances and Challenges, J. Phys. D: Appl. Phys. 2016, 49 413001.

2- W. Yi, C. Zhang, Q. Zhang, Y. Lu, L. Yi, X. Wang, Solid-State Nanopore/Nanochannel Sensing of Single Entities, Top Curr Chem (Z) 2023, 381, 13
